# Body Image Concerns and Psychological Distress in Adults with Hearing Aids: A Case-Control Study

**DOI:** 10.3390/audiolres15030062

**Published:** 2025-05-24

**Authors:** Enrico Apa, Silvia Ferrari, Daniele Monzani, Andrea Ciorba, Luca Sacchetto, Virginia Dallari, Riccardo Nocini, Silvia Palma

**Affiliations:** 1 Paediatric Audiovestibology Unit, Department of Neuroscience, Di Circolo Hospital, ASST dei Settelaghi, 21100 Varese, Italy; 2 Department of Biomedical, Metabolic and Neural Sciences, University of Modena and Reggio Emilia, 41121 Modena, Italy; 3 ENT, Department of Surgical Sciences, Dentistry, Gynaecology and Paediatrics, University of Verona, Borgo Roma Hospital, 37134 Verona, Italy; 4 ENT & Audiology Unit, Department of Neurosciences, University Hospital of Ferrara, 44124 Ferrara, Italy; 5 Department of Otolaryngology Head and Neck Surgery, Santa Maria delle Croci Hospital, AUSL della Romagna, 48121 Ravenna, Italy; 6 Audiology, Primary Care Department, AUSL of Modena, 41100 Modena, Italy

**Keywords:** stigma, hearing aids, body image, social adjustment

## Abstract

**Background/Objectives**: Hearing loss represents an important communication barrier which can lead to social isolation and can be a challenge for mental health status. It is generally accepted that hearing aid (HA) users can develop a stigma related to hearing loss despite the perceived benefits due to most modern technologies. Nevertheless, stigma toward HAs may persist even when patients have been well acclimatized to their use. This study aims to evaluate their experiences in everyday life, the underlying social aspects and the utility of psychometric multidimensional approach in skilled HA users. **Methods**: In total, 96 HA users and 85 normally hearing subjects were enrolled and asked to complete three psychometric questionnaires that investigated social functioning, body image perception, and psychological distress. HA users were additionally asked to fulfill a disease-specific survey, the International Outcome Inventory for Hearing Aids. The performance of the devices was also investigated by HA’s functional gain through free-field audiometry. **Results**: Even if auditory devices help with compensating the sensorial deprivation, patients often suffer from social anxiety, social phobia and body image concerns about their appearance while wearing HAs. **Conclusions**: This study discloses psychopathological factors associated with the HA experience that are surprisingly present after long satisfying HA use. Despite the benefits, the satisfaction and the daily use, HA users continue to be worried about body image and report more psychopathological distress in comparison to their normal hearing peers.

## 1. Introduction

Despite the advancements in HA technology, the stigma related to their use still represents a barrier for many potential users. The idea that a person possesses some features or characteristics impacting his/her social identity in a particular social context [1] is a current definition of stigma, a real obstacle to the effectiveness of many relevant public health programs or devices [2,3,4,5]. It has been recognized that stigma is context-specific and exists in the social environment of the person [1]. Stigma may also be related to the erroneous perception of the population affected by hearing impairment and/or wearing HAs [6]. A recent review suggests that stigma linked to hearing loss is related with aging and hearing loss, and may lead to social segregation, and negative self-perception [7].

Size and visibility of HAs and appearance were the reasons for many people to feel embarrassed while wearing them [8]. Many studies described HA stigma as one of the main reasons for hearing-impaired adults not wearing HAs, therefore leaving their hearing disability unaided [9,10].

This stigma-related phenomenon appeared more prevalent in young and middle-aged people than in the elderly [11] and may be influenced by gender [12]. Indubitably, the industry effort minimized the visibility of auditory devices and consequently reduced the negative attributes of the ‘hearing aids effect’ [9,10,11,12,13], a trend in which normal hearing individuals judge their peers wearing HAs as unattractive. Meanwhile, significant advances in technology have offered greater opportunities for hearing-impaired subjects than in the past, even if patients’ perception of HAs premium technology in real life is somewhat questionable [14]. Taken together, these data suggest that stigma associated with HAs should gradually decrease as the technology improves and HA visibility is reduced.

However, some adults with hearing loss still report negative feelings related to auditory devices [6,8,15] and stigma toward HAs as a social problem [16] and a cause for rejection/non-use [10,11,12,13,14,15,16,17]. In this regard, a recent study found that the stigma towards hearing loss rather than HAs was the major concern for hearing impaired adults and their families [18]. HAs can be considered as uncomfortable, unattractive, and, above all, associated with ageism [15,16], a process of systematic stereotyping and of discrimination against people simply because they are old. Not surprisingly, ageism is still identified as one of the main reasons for the non-use of HAs in many cases [17]. Concern about appearance has been confirmed by a recent study which suggests that younger and older adults may hold negative implicit attitudes towards both older and younger HA users [19]. Additionally, some individuals whose primary concern is appearance consider their auditory devices as an issue rather than a solution for their impairment, avoiding their use during societal events, thus preferring not to hear properly [20]. Body image concerns are now indicated as prevalent on a global scale [21], while they have been considered for a long time a risk factor for an individual’s identity and a deteriorated mental health [22]. Pressure to be attractive, strongly supported by advertising and social media, contributes to increasing this global risk [23]. Nevertheless, recent investigations seem to contradict previous results about body image concerns in HA users and indicate that both self-esteem and body image perception are improved by the use of auditory devices [24,25].

The present study takes origin from a previous experience at the University Hospital of Modena (Italy) that initially focused on body image perception and social phobia in patients with HAs and cochlear implants [26] and suggested the persistence of body image concerns and social phobia in patients with HAs, long after acclimatization.

Therefore, the aim of this investigation is the assessment of habitual HA users’ psychological distress, body image concerns and social perception to understand the dimension of the problems, if any, and to speculate about possible solutions. Randomly selected subjects wearing bilateral HAs, for at least one year, were recruited and asked to fill in four questionnaires: one (i) assessing psychological response and distress to physical illness and disability, another (ii) assessing body image-related discomfort, another (iii) considering the social and performance difficulties involved in phobic disorder and the last (iv) regarding the outcome domains for HA users. A control group of normal-hearing subjects, matched for demographic features, was administered the first 3 questionnaires and the answers from both groups were matched. The research question was to clarify how body image concern impacts on the decision to use HAs and how it correlates to psychological distress.

## 2. Materials and Methods

The present is a case–control study performed at the third level audiological out-patient center of the University Hospital of Modena. Randomly selected subjects wearing BTE (behind-the-ear) or ITC (in-the-ear) bilateral HAs, for at least one year, were invited to participate in the study. Cases were recruited while waiting for out-patient scheduled routine examinations or among hospital’s staff. Only subjects aged between 18 and 65 years were considered. Socio-demographic information was collected for all participants, including age, gender, marital status, educational qualification, weight and height in order to obtain the Body Mass Index (BMI). The two groups were matched for all these features.

Exclusion criteria were middle ear dysfunction or non-continuous use of hearing aids, presence of other oncological co-morbidities with a significant impact on functioning, language barriers (people not understanding/speaking Italian) or major psychiatric or neurological disorders that precluded the filling-out of questionnaires. No cases have been excluded only considering the degree of hearing loss or of the kind of auditory devices technologies.

### 2.1. Audiologic Assessment

Both cases and normal-hearing subjects underwent an audiologic examination that included pure tone audiometry and speech audiometry. Tympanometry and acoustic reflex testing were also performed to exclude subjects with middle ear dysfunction. A Madsen Zodiac device (Natus^®^ Medical Incorporated, Middleton, WI, USA) was used.

Pure tone audiometry was performed in a soundproof booth, testing each ear for air and bone conduction, at frequencies 0.25, 0.5, 1, 2, 4 and 8 kHz. A diagnostic audiometer (MADSEN Astera 1066 type, GN Otometrics A/S, Natus Medical Incorporated, Middleton, WI, USA) was used. TDH-39 headphones were supplied to depict the air conduction hearing threshold.

The mean pure tone average (PTA) was calculated as an average at 0.5, 1, 2 and 4 kHz, for both ears. In the case of asymmetric hearing impairment, the PTA of the better-ear was the base for classification of the hearing gloss entity, according to the American Speech-Language-Hearing Association [27]. In detail, the hearing loss was considered mild when PTA ranged between 26 and 40 dB HL, moderate with PTA ranged between 41 and 55 dB HL, and moderately severe when PTA ranged between 56 and 70 dB HL. For the cases, type of HA, years of hearing aid use and daily use (expressed as hours) were additionally collected and considered. HA’s functional gain was tested by sound-field measurement as previously described [28]. Speech audiometry was performed using 5 lists of 20 two-syllable (spondees) recorded words which were administered in both aided and unaided conditions with competing background noise. The total amount of spondees correctly repeated was computed as a percentage and termed Words Recognition Score (WRS). Single patient’s amplification and other technical parameters of the hearing aids were not assessed as variables in this study.

All control group subjects had a bilateral hearing threshold within 20 db HL and normal tympanometry.

### 2.2. Body Mass Index

The Body Mass Index (BMI) [29] was computed according to the following formula: Weight in kg/Height in m^2^. BMI is generally used to categorize people on body mass and height. According to this index, adults are referred to as underweight (under 18.5 kg/m^2^), normal weight (18.5 to 24.9), overweight (25 to 29.9), and obese (30 or more). In the framework of this study, the BMI was adopted as a statistical measurement for groups since it is generally accepted that it is positively associated with body dissatisfaction in both genders [30].

### 2.3. Questionnaires

After providing informed consent, participants filled in the validated Italian versions of the following self-report questionnaires. The development and validation processes of the questionnaires are indicated in the published related articles (all cited in this paper).

- **The Brief Symptom Inventory (BSI)** [31,32]. BSI assesses the psychological response and distress to physical illness and disability. In detail, it consists of 53 items that measure somatization (SOM), obsessive-compulsivity (OBS), interpersonal sensitivity (INS), depression (DEP), anxiety (ANX), hostility (HOS), phobic anxiety (PHOB), paranoid ideation (PAR), and psychoticism (PSY). The nine dimensions can be summed up to reflect three global indices: Global Severity Index (GSI), Positive Symptom Total (PST) and Positive Symptom Distress Index (PSDI). The GSI is calculated using the sums for the nine symptom dimensions and dividing by the total number of items. It is a sensitive indicator of the overall level of distress. The PST is a count of all the items with non-zero responses and reveals the absolute number of endorsed symptoms. The PSDI is the sum of the values of the items receiving non-zero responses divided by the PST. It provides information about the average level of distress the respondent experiences.
  The items are then rated, from 0 (‘not at all’) to 4 (‘extremely’), ranking the distress intensity in the last week.
- **The Body Uneasiness Test (BUT)** [33]. This instrument is a psychometric assessment of body image-related discomfort. In the present study, the **BUT-A** scale was used, containing 34 items that can be divided into the following categories: fear of being or becoming fat (Weight phobia—WP), concerns associated with the physical appearance (Body Image Concerns—BIC), body image related to avoidance conduct (Avoidance—A), compulsive evaluation of physical features (Compulsive Self-Monitoring—CSM) and estrangement body feelings (Depersonalization—D). Each item is rated on a six-point Likert-type scale ranging 0–5 (from ‘never’ to ‘always’) and high rates indicate greater bodily discomfort. The BUT-B, which looks at specific worries about particular body parts or functions, was not used in the present study.
- **The Liebowitz Social Anxiety Scale (LSAS)** [34,35]. It is a scale that considers the social and performance difficulties involved in phobic disorder. The LSAS consists of 24 items, 11 of which explore social interaction and 13 actual situations. For each item, the amount of fear and avoidance related to the given scenario must be indicated, with a rating from 0 to 3 on a Likert scale. The total score expressed on 48 answers indicates increasing social anxiety and can be divided into the two fear (F) and avoidance (A) subscales, which in each case are composed of social interaction (S-F and S-Av) and performance domains (P-F and P-A). According to the authors, a total score ranging 30–50 indicates mild social anxiety, whereas scores 50–65 or 65–80 are consistent with moderate and marked social anxiety.
- **The International Outcome Inventory for Hearing Aids (IOI-HA)**, Italian version, was proposed to the case group [36,37]. It consists of seven items regarding the outcome domains of specific item tests for assisted hearing device carriers, including the following subscales: Daily Use (USE), Benefit (BEN), Residual Activity Limitation (RAL), Satisfaction (SAT), Residual Participation Restrictions (RPR), Impact on others (IOTH), and Quality of life (QOL). Each item has five response choices, on a Likert scale, ranging from 1 (least favorable) to 5 (most favorable). Therefore, the score ranges from 7 to 35. The higher the total score of the scale, the higher the degree of patients’ satisfaction with their hearing aids. The Cronbach’s α on the original version was 0.78 and this instrument was also later translated in Italian [38,39].

### 2.4. Statistical Analysis

The SPSS^®^ (Statistical Package for Social Sciences-version 26.0) has been used for the data statistical evaluation. The Shapiro–Wilk test detected an abnormal distribution for all the variables (W = 0.643–0.941; *p* > 0.05), excluding age, BMI and IOI-HA (W = 0.773–0.809; *p* < 0.05). Pearson’s chi-square test was performed to test the hypothesis that epidemiological variables between case and control groups were equal. The U Mann–Whitney Test was adopted for age, BMI and IOI-HA, while the Independent *t*-test f was used for the other continuous variables.

The same tests were applied for the BSI, BUT-A and LSAS scales scores. The sample was divided according to age into two subgroups: one aged 18–40 years and one aged 40–65 years. This arbitrary limit should avoid dispersion of the cases due to the relatively small number of subjects enrolled.

Finally, the one-way ANOVA with Bonferroni post hoc analysis was used to detect any differences in IOI-HA, BSI, BUT-A and LSAS in the case group according to the wearing of ITE or BTE hearing aids. The statistical significance was considered when the *p*-value was <0.05. A further statistical analysis was performed to test internal consistency reliability of the IOI-HA by calculating the Cronbach’s α coefficient [40]. This measure is used to determine the extent to which items within a test or survey are consistent with each other, meaning they evaluate the same concept.

### 2.5. Ethical Considerations

Ethical principles according to the Declaration of Helsinki on human experimentation were followed. The study was approved by the institutional ethical committee (protocol code CE 76/2007). Informed consent was obtained by each participant.

## 3. Results

Ninety-six subjects wearing bilateral hearing aids were enrolled in the study, while eighty-five subjects with normal hearing matched for all epidemiological features were recruited as the control group (see also Table 1).

Mean PTA was 20.87 dB HL (12–25; SD ± 3.22) in the control group and 47.11 dB HL (34–63; SD ± 6.68) in HA users. In detail, 16 subjects (16.7%) presented a mild hearing loss, 69 (71.9%) a moderate hearing loss and 11 (11.4%) a moderately severe hearing loss.

Regarding BSI scores, in the HA group, significantly higher GSI and PST were detected (see also Table 2). Higher mean of SOM, DEP, ANX, PHOB, PSY and INS were observed, whereas no significant difference resulted concerning PAR, HOS and OBS.

Results of BUT-A and LSAS in both groups are presented in Table 3 and Table 4. Significantly higher values were observed in HA users concerning the two questionnaires and all their subscales. In particular, the LSAS total score was consistent with mild social anxiety in the case group, whereas this aspect did not emerge in controls (Table 4).

The case group consisted of 22 subjects (17 BTE users and 5 ITE users) aged 18–40 years and 74 aged 41–65 years (44 and 30 subjects were BTE and ITE wearers, respectively). No significant differences were observed according to HA type in the different age subgroups (*p* = 0.127). Table 5 shows the BSI, BUT-A, and LSAS scales results. In detail, in older subjects, significantly higher avoidance and depersonalization were detected using the BUT-A. No other significant difference was observed.

Concerning the IOI-HA, mean scores of single items and the total score are reported in Table 6. Cronbach’s α was 0.84, higher than the generally accepted 0.80 “cutoff value” for general research purposes [39]. Significantly higher RAL and IOTH were present in 41–65 year old subjects. No significant moderate, strong or very strong correlations were observed between PTA in aided condition and IOI-HA, BSI, BUT-A, or LSAS (Spearman RHO = −0.194–0.091; *p* > 0.05).

**Figure 1:**
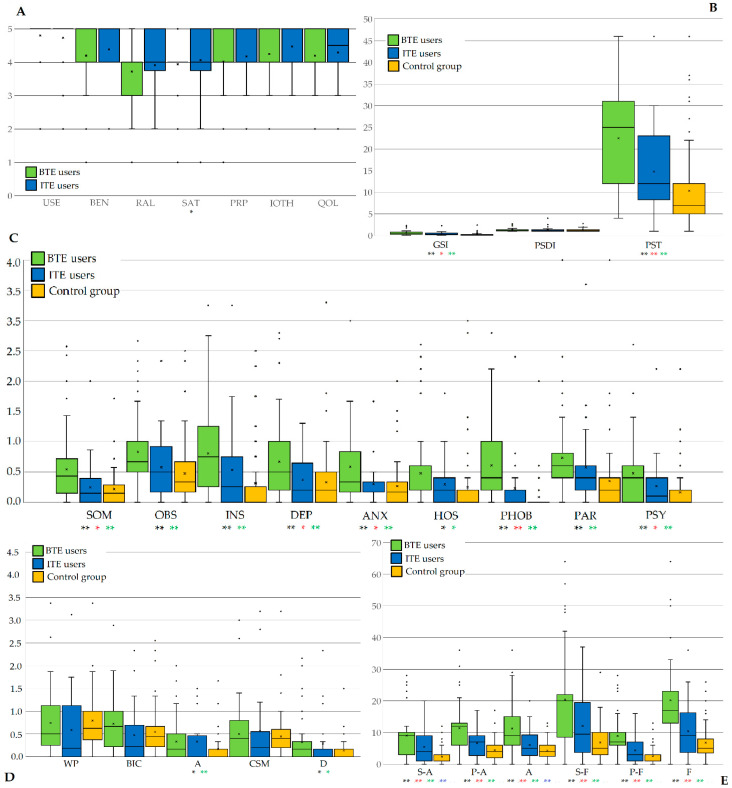
Results considering the type of HAs. (**A**) Results for IOI-HAs. (**B**) Results for BSI. (**C**) Results for BSI’s domains. (**D**) Results for the BUT-A scale. (**E**) Results for LSAS. Each box is included between the first and third quartile; values differing more than 1.5 of Inter Quartile Range (IQR) upwards or downwards are considered as possible anomalies and represented with °. *p* values under 0.05 (*) or under 0.005 (**) are displayed in black for Independent *t*-test or one-way ANOVA, or in red, green or blue for Bonferroni post hoc analysis between BTE and ITE users, BTE users and control group or ITE users and control group, respectively. ITE (in-the-ear hearing aids), BTE (behind-the-ear hearing aids), USE (Daily Use), BEN (Benefit), RAL (Residual Activity Limitation), SAT (Satisfaction), RPR (Residual Participation Restrictions), IOTH (Impact on others), QOL (Quality of life), GSI (Global Severity Index), PST (Positive Symptom Total), PSDI (Positive Symptom Distress Index), SOM (somatization), OBS (obsessive-compulsivity), INS (interpersonal sensitivity), DEP (depression), ANX (anxiety), HOS (hostility), PHOB (phobic anxiety), PAR (paranoid ideation), PSY (psychoticism), WP (Weight phobia), BIC (Body Image Concerns), A (Avoidance), CSM (Compulsive Self-Monitoring), D (Depersonalization), S-A (avoidance of social interaction), P-A (avoidance of performance avoidance), A (avoidance), S-F (social interaction fear), P-F (performance fear), F (fear).

Regarding HAs, the case group was composed of 61 BTE and 35 ITE wearers. The years of use were 12.26 (1–38; SD ± 10.86) and 10.74 (1–40; SD ± 11.27), respectively (*p* = 0.984). The mean PTA was 48.03 dB HL (34–63; SD ± 7.36) in the former and 45.56 dB HL (35–54; SD ± 5.05) in the latter. In unaided condition, WRS was 63.49 (35–76; ± 9.39) and 67.79 (56–76; ± 4.32) in BTE and ITE wearers, respectively, whereas in aided condition, WRS was 83.13 (55–100; ± 9.92) and 85.82 (78–100; ± 6.42). Considering subjects with BTE and ITE, the differences were significant for PTA and WRS in unaided condition (*p* = 0.038; *p* = 0.003) and not significant for WRS in aided condition (*p* = 0.158). Excluding the satisfaction item (*p* = 0.037), no significant differences were observed considering the IOI-HA (Figure 1A).

Figure 1 shows differences among BTE users, ITE users and controls. Excluding PSDI, for The Brief Symptom Inventory and its domains, significant differences resulted with the one-way ANOVA test (Figure 1B,C). In particular, GSI and PST resulted in significantly higher sores in BTE users compared to controls (*p* = 0.017, *p* = 0.002) and ITE users (*p* = 0.000, *p* = 0.000). Similarly, BTE users presented significantly higher values of SOM, DEP, ANX, PSY, and PHOB. For OBS, INS, HOS and PAR, BTE users presented significantly higher values only when compared to the control group, whereas no significant differences resulted between BTE and ITE users. No further analysis was performed concerning technical specifications (sourced from manufacturers) of devices because they extensively vary from one type to another and this could provoke an excessive dispersion of data.

Figure 1D shows that, for the BUT-A scale, significant differences were detected for avoidance (*p* = 0.017) and depersonalization domains (*p* = 0.022). Bonferroni post hoc analysis confirmed the significance only for BTE users compared to the control group (*p* = 0.001, *p* = 0.025). In contrast, The LSAS presented significant differences between BTE and ITE users and between BTE users and the control group (*p* = 0.000). In addition, significantly higher values of S-A (*p* = 0.010) and A were observed in ITE users versus the control group (*p* = 0.037) (Figure 1E).

Regarding the IOI-HA mean scores of single items and the total score, Cronbach’s α was 0.84, which is higher than the generally accepted 0.80 “cutoff value” for general research purposes [39]. Mean scores of the Use, Benefit and Satisfaction questions were near the highest possible score.

## 4. Discussion

The purpose of the study was to evaluate the underlying psycho-social factors that affect the use of HAs. More specifically, global psychopathology, social anxiety and avoidance, and discomfort with self-body perception were evaluated. Results demonstrated that HA users generally declared to be satisfied with their devices and to find them beneficial, as documented by high scores recorded by the IOI-HA. In contrast, this study documented that cases report higher levels of distress in comparison to controls in terms of general psychopathology.

Actually, cases scored higher than controls in all the subscales of the BSI excluding hostility, paranoid ideation and obsessive-compulsivity. The difference was particularly evident for anxiety, phobic anxiety, somatization and interpersonal sensitivity. It should be observed that this study design (cross-sectional) does not allow quantifying if a higher level of anxiety is due to HA use or to the higher prevalence of anxiety amongst people with hearing loss in relation to the general population [41], or both. Similarly, hearing status itself is negatively associated with depression and somatization in young and middle-aged adults [42] and a direct causative effect of HAs on this domain is questionable. Furthermore, symptoms of depression in hearing-impaired adults seem to be more linked to the fear of perceiving a negative evaluation by others, that is, stigma [43]. This investigation does not suggest that patients age was a relevant factor in relation to psychological distress or to social adjustment. It also excludes that the degree of hearing loss is correlated to the aforementioned variables. A more intriguing question arises from the higher score of interpersonal sensitivity. Interpersonal sensitivity contributes to more effective communication, as it allows better understanding of non-verbal signals, interpreting the emotions behind the words [44]. Non-verbal communication (i.e., body language or the voice tone) plays a crucial role in interpersonal sensitivity. It could be suggested that suboptimal speech perception in HA users (especially in noisy environments) forces them to develop a great series of evaluations of non-verbal signals to facilitate meaningful connections with others.

Individuals with HAs, in our sample, were more frequently involved in disorders related to the perception of their body image than those in the control group. Body image is a mental representation based on an individual’s appearance, attractiveness and physical health [45]. Body image concerns are the results of perceptual inaccuracy, cognitive distortion, affective reactions and attitudinal and behavioral aspects [46].

The BUT-A scores revealed that the HA users frequently adopt avoidant behaviors associated with their self-image and report detachment from their own body (depersonalization). It is also known that subjective dissatisfaction of self-body potentially influences perceived stigma independently from objective body shape or the type of condition causing the stigma, for example, hearing loss or/and use of hearing aids [47]. These results seem to confirm the findings of a previous study which revealed that self-image is one of the most concerns in getting used to hearing aids [48].

The LSAS confirmed that HA users are affected by higher levels of fear and avoidance associated with social phobia than controls. Daily and common situations such as talking and using the telephone in public, eating in a pub or being the center of attention can promote, in HA users, a sense of discomfort and embarrassment that, in turn, leads to avoidance behaviors and a poor personal and social adjustment.

It should be noted that both psychological and body image distress and social anxiety were higher in BTE users than in subjects wearing ITE devices, despite speech perception in aided condition not being statistically different. A possible explanation for these differences is that despite modern BTE hearing aids being smaller and slighter than older models, they are still visible, especially for people with short haircuts. On the other hand, ITE are often discrete devices that are hardly visible around the outer ear. HA appearance was one of the most important factors for choosing one type over another [49]. No significant differences were observed according to HA type in the different age subgroups.

Despite a recent investigation showing that using HAs for six weeks induced a positive effect on hearing-impaired patients when wearing HAs [50], this study clearly indicates that preoccupation and dissatisfaction towards bodily appearance persists after a long time of hearing aid use. Important advances among technological and aesthetic features for HAs have been reported in recent years, ensuring a significant sound quality improvement and the elimination of background noise.

This study also confirms the results of another recent experiment showing that HA users appreciate current technological advances in terms of benefit and satisfaction but express a need for improvements. Actually, it has been reported that physical aesthetics is a key point for these improvements [51], suggesting a persistent preoccupation with HA stigma and the need for changes in societal cultures and perceptions [52]. The results of this study suggest educating ENT residents and audiologists to counteract the psychopathological frailty associated with HA prescription and use, also based on Artificial Intelligence [53]. Hearing loss stigma appears to be pervasive across age and gender with distinctions that have implications for interventional development [54]

Measures should be adopted by the community to reduce the stigma consequences, starting with education campaigns. By promoting the consideration that hearing loss is a common part of aging or can occur at any age, the perception of HA concern can change. For example, celebrities or professionals with hearing aids can help reduce the stigma perception, especially among young people. Reframing the concept of disability to a broader, more inclusive view of diversity can also help. The idea that everyone, at some point in their life, may need some form of assistance or adaptation (like glasses or hearing aids) can help the experience feel more common and less stigmatized. If HAs become as ubiquitous and unobtrusive as eyeglasses, the stigma could be gradually reduced. Furthermore, personalized options, such as customizing HAs to the individual’s preferences can make the HA experience more comfortable and empowering.

Reducing the stigma around HAs requires a complex approach involving technological innovation, societal change, and individual empowerment. It is evident that technological innovation is always ongoing and that families and professionals are improving their approach to the questions raised by HAs. Societal change appears to be a slow process that takes time, but also plays a crucial role [52]. In the “seventies”, people experienced stigma around wearing glasses, and then glasses became part of the “fashion world” and the related stigma gradually disappeared within some years. There are some signs that this could also happen in the “world of hearing aids” [55].

The main limitation of the study is its nature, as it has not been possible to distinguish if higher levels of anxiety are due to hearing aid use or to the higher prevalence of anxiety among people with hearing impairment. Self-report biases and the lack of data on HA non-users with hearing loss are also other limitations. Furthermore, distinctions were not possible between the experiences of those with congenital versus acquired hearing loss.

## 5. Conclusions

Despite Benefit, Satisfaction and Daily Use of hearing aids, hearing-impaired HA users continue to perceive concerns about their body image and to report more psychopathological distress in relation to their normal hearing peers. Poor mental health and social isolation are still associated with HA use, regardless of the type of auditory devices (ITE and BTE). This study confirms that concerns about body image and appearance are a multidimensional construct encompassing beliefs, feelings and thoughts, not related to the technological and aesthetic evolutions of auditory devices. HA users deserve a multidimensional clinical approach and enlarged diagnostic skills in order to satisfy as many aspects of their psychological profile and their social behavior

Future studies are necessary to expand the application fields of this approach in order to evaluate other possible implications in the clinical setting and to resolve the related stigma perception.

## Figures and Tables

**Table 1:** Demographic characteristics of the sample size (case group versus control group).

	Case Group (*n* = 96)	Control Group (*n* = 85)	Significance ^a,b^
Age	57.31 (36–65; SD ± 9.34)	58.39 (33–65; SD ± 9.21)	0.354 ^a^
Gender	46 (47.8%) females 50 (52.2%) males	43 (50.6%) females 42 (49.4%) males	0.720 ^b^
Marital Status	56 (58.3%) singles 40 (41.7%) married	53 (62.4%) singles 32 (37.7%) married	0.581 ^b^
Educational qualification	54 (56.2%) primary school 21 (21.9%) high school 21 (21.9%) university	46 (54.1%) primary school 23 (27.1%) high school 16 (18.8%) university	0.741 ^b^
BMI	23.70 (18.37–38.22; ± SD 4.07)	24.73 (19.53–30.59; SD ± 3.22 SD	0.053 ^a^

^a^ U Mann–Whitney test, ^b^ Pearson’s chi-square test). SD (Standard deviation), BMI (Body Mass Index = Weight in kg/Height in m^2^).

**Table 2:** Results of the Brief Symptom Inventory (BSI) through ^a^ Independent *t*-test and ^b^ Pearson’s chi-square test, *p* < 0.05 = (*), *p* < 0.005 = (**). GSI (Global Severity Index), PST (Positive Symptom Total), PSDI (Positive Symptom Distress Index), SOM (somatization), OBS (obsessive-compulsivity), INS (interpersonal sensitivity), DEP (depression), ANX (anxiety), HOS (hostility), PHOB (phobic anxiety), PAR (paranoid ideation), PSY (psychoticism), and SD (Standard deviation).

	Case Group (*n* = 96)	Control Group (*n* = 85)	Significance ^a,b^
GSI	0.53 (0.06–2.33; SD ± 0.50)	0.28 (0.19–2.42; SD ± 0.36)	** 0.001 ^a^
PSDI	1.33 (1.00–4.00; SD ± 0.51)	1.27 (1.00–2.78; SD ± 0.33)	0.118 ^a^
PST	19.61 (0.00–46; SD ± 11.96)	10.32 (1.00–46; SD ± 9.28)	** 0.000 ^a^
SOM	0.44 (0.00–2.57; SD ± 0.54)	0.22 (0.00–1.71; SD ± 0.29)	** 0.001 ^a^
OBS	0.73 (0.00–2.67; SD ± 0.58)	0.47 (0.00–2.50; SD ± 0.51)	0.067 ^a^
INS	0.70 (0.00–3.25; SD ± 0.75)	0.31 (0.00–2.50; SD ± 0.53)	** 0.002 ^a^
DEP	0.55 (0.00–2.80; SD ± 0.59)	0.32 (0.00–3.33; SD ± 0.50)	* 0.026 ^a^
ANX	0.48 (0.00–3.00; SD ± 0.59)	0.26 (0.00–2.00; SD ± 0.37)	** 0.000 ^a^
HOS	0.41 (0.00–2.60; SD ± 0.56)	0.25 (0.00–3.00; SD ± 0.49)	0.103 ^a^
PHOB	0.47 (0.00–2.80; SD ± 0.60)	0.09 (0.00–2.00;SD ± 0.25)	** 0.000 ^a^
PAR	0.70 (0.00–4.00; SD ± 0.66)	0.35 (0.00–4.00; SD ± 0.57)	0.130 ^a^
PSY	0.40 (0.00–2.60; SD ± 0.53)	0.16 (0.00–2.20; SD ± 0.33)	** 0.000 ^a^

**Table 3:** Results of BUT-A scale through ^a^ Independent *t*-test, ^b^ Pearson’s chi-square test, *p* < 0.05 = (*), *p* < 0.005 = (**). WP (Weight phobia), BIC (Body Image Concerns), A (Avoidance), CSM (Compulsive Self-Monitoring), D (Depersonalization), and SD (Standard deviation). Significantly higher values were observed in HAs users.

	Case Group (*n* = 96)	Control Group (*n* = 85)	Significance ^a,b^
WP	0.68 (0.0–3.38; SD ± 0.74)	0.80 (0.0–4.38; SD ± 0.69)	* 0.025 ^a^
BIC	0.63 (0.0–2.89; SD ± 0.68)	0.55 (0.0–2.55; SD ± 0.52)	** 0.001 ^a^
A	0.34 (0.0–2.00; SD ± 0.47)	0.17 (0.0–1.67; SD ± 0.32)	** 0.001 ^a^
CSM	0.52 (0.0–3.20; SD ± 0.75)	0.45 (0.0–3.20; SD ± 0.45)	** 0.000 ^a^
D	0.31 (0.0–2.33; SD ± 0.52)	0.14 (0.0–1.50; SD ± 0.28)	** 0.000 ^a^

**Table 4:** Results of LSAS through ^a^ Independent *t*-test, ^b^ Pearson’s chi-square test, ), *p* < 0.005 = (**). S-A (avoidance of social interaction), P-A (avoidance of performance avoidance), A (avoidance), S-F (social interaction fear), P-F (performance fear), F (fear), LSAS (Liebowitz Social Anxiety Scale), and SD (Standard deviation). The LSAS total score was consistent with mild social anxiety in the case group.

	Case Group (*n* = 96)	Control Group (*n* = 85)	Significance ^a,b^
S-A	7.72 (0–28; SD ± 7.72)	2.40 (0–12; SD ± 2.66)	** 0.000 ^a^
P-A	9.64 (0–36; SD ± 9.64)	4.45 (0–17; SD ± 3.34)	** 0.000 ^a^
A	17.35 (0–64; SD ± 17.35)	6.85 (0–29; SD ± 5.69)	** 0.000 ^a^
S-F	7.28 (0–28; SD ± 7.28)	2.52 (0–13; SD ± 3.07)	** 0.001 ^a^
P-F	9.35 (0–36; SD ± 9.35)	4.32 (0–13; SD ± 2.73)	** 0.000 ^a^
F	16.64 (0–64; SD ± 16.64)	6.84 (0–26; SD ± 5.52)	** 0.000 ^a^
LSAS	33.99 (0–128; SD ± 33.99)	13.68 (0–52; SD ± 10.67)	** 0.000 ^a^

**Table 5:** Results of the BSI, BUT-A, LSAS scales for the given age groups.

	18–40 Years Old (*n* = 22)	41–65 Years Old (*n* = 74)	Significance ^a,b^
GSI	0.60 (0.11–2.34; ±0.56)	0.51 (0.06–2.26; ±0.48)	0.465 ^a^
PSDI	1.25 (1.00–2.70; ±0.43)	1.36 (1.00–4.00; ±0.53)	0.390 ^a^
PST	22.45 (6–46; ±13.51)	18.77 (1–46; ±11.41)	0.206 ^a^
WP	0.59 (0.0–1.62; ±0.53)	0.71 (0.0–3.38; ±0.79)	0.512 ^a^
BIC	0.66 (0.0–1.22; ±0.47)	0.62 (0.0–2.89; ±0.73)	0.826 ^a^
A	0.12 (0.0–0.50 ±0.18)	0.41 (0.0–2.00; ±0.50)	* 0.011 ^a^
CSM	0.36 (0.0–1.40. ±0.39)	0.57 (0.0–3.20; ±0.82)	0.270 ^a^
D	0.11 (0.0–0.50; ±0.17)	0.37 (0.0–2.33; ±0.57)	* 0.036 ^a^
S-A	8.50 (2–21; ±5.09)	7.49 (0–28; ±7.09)	0.534 ^a^
P-A	9.55 (3–21; ±4.76)	9.66 (0–36; ±7.61)	0.946 ^a^
A	18.05 (5–42; ±9.81)	17.15 (0–64; ±14.44)	0.786 ^a^
S-F	7.95 (2–17; ±3.44)	7.08 (0–28; ±6.43)	0.543 ^a^
P-F	9.41 (4–16; ±4.61)	9.34 (0–36; ±7.64)	0.967 ^a^
F	17.36 (7–33; ±7.30)	16.42 (0–64; ±13.86)	0.760 ^a^
LSAS	35.41 (12–75; ±16.48)	33.57 (0–128; ±27.78)	0.769 ^a^

^a^ Independent *t*-test, ^b^ Pearson’s chi-square test, *p* < 0.05 = (*). GSI (Global Severity Index), PST (Positive Symptom Total), PSDI (Positive Symptom Distress Index), WP (Weight phobia), BIC (Body Image Concerns), A (Avoidance), CSM (Compulsive Self-Monitoring), D (Depersonalization), S-A (avoidance of social interaction), P-A (avoidance of performance avoidance), A (avoidance), S-F (social interaction fear), and P-F (performance fear), F (fear).

**Table 6:** International Outcome Inventory (IOI-HA) results.

	Case Group (*n* = 96)	18–40 Years Old (*n* = 22)	41–65 Years Old (*n* = 74)	Significance ^a^
USE	4.78 (2–5; ±0.57)	4.82 (4–5; ±0.40)	4.77 (2–5; ±0.61)	0.729 ^a^
BEN	4.27 (1–5; ±1.04)	4.41 (3–5; ±0.67)	4.23 (1–5; ±1.14)	0.481 ^a^
RAL	3.77 (1–5; ±1.04)	4.05 (4–5; ±0.21)	3.69 (1–5; ±1.17)	** 0.015 ^a^
SAT	3.98 (1–5; ±0.87)	4.14 (4–5; ±0.35)	3.93 (1–5; ±0.97)	0.135 ^a^
RPR	4.07 (1–5; ±0.93)	4.09 (3–5; ±0.75)	4.07 (1–5; ±0.98)	0.914 ^a^
IOTH	4.33 (2–5; ±0.69)	4.64 (4–5; ±0.49)	4.24 (2–5; ±0.72)	* 0.005 ^a^
QOL	4.23 (2–5; ±0.76)	4.18 (3–5; ±0.59)	4.24 (2–5; ±0.81)	0.741 ^a^
TOT	29.44 (13–35; ±4.31)	30.32 (27–35; ±1.59)	29.18 (13–35; ±4.81)	0.084 ^a^

^a^ Mann–Whitney U test, *p* < 0.05 = (*), *p* < 0.005 = (**). USE = daily use in hours, BEN = benefit, RAL = residual activity limitations, SAT = satisfaction, RPR = residual participation restrictions, IOTH = impact on others and, and QOL = quality of life.

## Data Availability

The data presented in this study are available on request from the corresponding author, due to privacy restrictions.
